# TRPM6 is Essential for Magnesium Uptake and Epithelial Cell Function in the Colon

**DOI:** 10.3390/nu10060784

**Published:** 2018-06-18

**Authors:** Francesca Luongo, Giuseppe Pietropaolo, Mathieu Gautier, Isabelle Dhennin-Duthille, Halima Ouadid-Ahidouch, Federica I. Wolf, Valentina Trapani

**Affiliations:** 1 Istituto di Patologia Generale, Università Cattolica del Sacro Cuore, Fondazione Policlinico Universitario “Agostino Gemelli” IRCCS, I-00168 Rome, Italy; francesca.luongo@unicatt.it (F.L.); giuseppepietrop1993@libero.it (G.P.); 2 Laboratoire de Physiologie Cellulaire et Moléculaire-EA4667, UFR Sciences, Université de Picardie Jules Verne, F-80039 Amiens, France; mathieu.gautier@u-picardie.fr (M.G.); isabelle.dhennin-Duthille@u-picardie.fr (I.D.-D.); ha-sciences@u-picardie.fr (H.O.-A.)

**Keywords:** cell migration, cell proliferation, intestine, ion imaging, magnesium channel, magnesium homeostasis, MagT1, NS8593, TRPM7, wound healing

## Abstract

Intestinal magnesium (Mg) uptake is essential for systemic Mg homeostasis. Colon cells express the two highly homologous transient receptor potential melastatin type (TRPM) 6 and 7 Mg^2+^ channels, but their precise function and the consequences of their mutual interaction are not clear. To explore the functional role of TRPM6 and TRPM7 in the colon, we used human colon cell lines that innately express both channels and analyzed the functional consequences of genetic knocking-down, by RNA interference, or pharmacological inhibition, by NS8593, of either channel. TRPM7 silencing caused an increase in Mg^2+^ influx, and correspondingly enhanced cell proliferation and migration, while downregulation of TRPM6 did not affect significantly either Mg^2+^ influx or cell proliferation. Exposure to the specific TRPM6/7 inhibitor NS8593 reduced Mg^2+^ influx, and consequently cell proliferation and migration, but Mg supplementation rescued the inhibition. We propose a model whereby in colon cells the functional Mg^2+^ channel at the plasma membrane may consist of both TRPM7 homomers and TRPM6/7 heteromers. A different expression ratio between the two proteins may result in different functional properties. Altogether, our findings confirm that TRPM6 cannot be replaced by TRPM7, and that TRPM6/7 complexes and TRPM6/7-mediated Mg^2+^ influx are indispensable in human epithelial colon cells.

## 1. Introduction

Magnesium (Mg) is involved in virtually all major metabolic and signaling pathways in the cell, and disturbances of Mg homeostasis accompany a variety of diseases [1]. Despite recent advancements in understanding the critical role of Mg in health and disease, the molecular mechanisms governing cellular and systemic Mg balance remain debated. Systemic Mg homeostasis primarily depends on the concerted actions of the intestine responsible for Mg uptake from food, and the kidneys, that regulate urinary Mg excretion [1]. Classical physiological experiments with different animal species have identified two Mg transport systems in the intestinal epithelium: An active transcellular and a passive paracellular pathway [2,3]. The paracellular pathway, which is driven by the electrochemical gradient, is responsible for bulk Mg absorption and takes place mostly in the small intestine, whereas fine-tuning occurs in the cecum and colon via transcellular transport. The transcellular route consists of apical Mg entry, mediated by Mg permeable channels, and a basolateral extrusion step involving putative Na^+^/Mg^2+^ exchangers.

In the last two decades, several proteins have been proposed to facilitate Mg transport [4], but the biological roles of most of them continue to arouse dispute. The best-characterized transporters are the transient receptor potential melastatin (TRPM) 6 and 7 cation channels. Both TRPM6 and TRPM7 contain a transmembrane TRP channel segment fused to a cytosolic α-type serine/threonine protein kinase domain, but the functional relationship and the potential interplay between the channel and kinase moieties are still debated [5]. TRPM7 is ubiquitously expressed in human tissues and has been proposed as an indispensable cellular Mg entry pathway since TRPM7-deficient cell lines have intracellular Mg deficiency and severe cell growth defects [6,7]. The highly homologous TRPM6 channel has a more restricted expression: The highest levels are found in the distal convoluted tubule of the kidney and in the distal small intestine and colon, in murine as well as human tissues [8,9]. Interestingly, TRPM6 expression in the kidney and intestine is regulated by dietary Mg availability [8,9,10,11]. The critical role of TRPM6 for systemic Mg homeostasis became evident when loss-of-function mutations in the TRPM6 gene were discovered in patients with a rare form of hereditary hypomagnesemia (hypomagnesemia with secondary hypocalcemia, HSH) [12,13]. Although the etiology of low Mg levels in HSH was initially ascribed to renal Mg wasting, recent findings challenged this view suggesting that a defect in the intestinal Mg uptake might be of primary relevance [14]. Consequently, it is crucial to identify the key molecular players of intestinal Mg absorption and their regulation.

It is intriguing that transporting epithelia such as colon mucosa express both TRPM6 and TRPM7. Pioneering work showed that the two proteins are not redundant, and the functional ion channel at the plasma membrane is a multimeric complex consisting of either TRPM7 homotetramers or TRPM6/7 heterotetramers, each possessing different biophysical properties [15,16,17]. However, most findings were derived from heterologous expression models and resulted in considerable controversy on the relationship between the two channels and their exact functional role (for an up-to-date review, see Reference [5]). Recent data from our group seem to corroborate the view that TRPM6, rather than TRPM7, might have a central role in the colon; indeed, we showed that dietary Mg exerts a protective effect on colonic mucosa by upregulating TRPM6 expression in an in vivo model [18]. In the present work, we sought to scrutinize the exact role of each of the two sister channels in the colon. We decided to use human colon cell lines that innately express both channels, and, therefore, may well epitomize the physiological context. In our model cell lines, we dissected the contribution of each channel, by specifically RNA interference (RNAi) silencing either of them, and analyzing the functional consequences in terms of magnesium influx, proliferation, and migration. We present data suggesting that an alteration in the ratio between differently assorted tetramers might constitute a flexible way to modulate cation influx and affect related signaling pathways.

## 2. Materials and Methods

### 2.1. Cell Culture

Human colon carcinoma HT29, HCT116, and RKO cells were routinely grown in Dulbecco’s modified Eagle’s medium (DMEM) supplemented with 10% fetal bovine serum (FBS), 2 mM glutamine, 100 U/mL penicillin, and 100 μg/mL streptomycin in a 5% CO_2_ humidified atmosphere at 37 °C. Human colon carcinoma Caco-2 cells were grown in the same medium supplemented with 20% FBS. To assess cell proliferation in conditions of different Mg availability, Mg-free DMEM (Invitrogen) was supplemented as for routine culture plus the desired amounts of MgSO_4_. To obtain a transient downregulation of TRPM7, we used a short interfering RNA (siRNA) targeting the nucleotide sequence 5′-GTCTTGCCATGAAATACTC-3′ (Dharmacon Research Inc., Chicago, IL, USA). For silencing TRPM6, predesigned siRNAs against human TRPM6 were purchased from Qiagen. Specific siRNAs were transfected into cells (50 ng per 400,000 cells) using HiPerFect Transfection Reagent (Qiagen Srl., Milan, Italy) following the manufacturer’s protocol. Non-silencing, scrambled sequences were used as controls (CTRL). NS8593 hydrochloride salt was purchased from Sigma Aldrich; a stock solution was made in dimethyl sulfoxide (DMSO) and stored in aliquots at −20 °C. To assess proliferation, cells were counted on a Thoma chamber in duplicate samples at given time points.

### 2.2. Western Blot

Cells were lysed in radioimmunoprecipitation assay (RIPA) buffer (50 mM Tris, pH = 8, 150 mM NaCl, 1 mM ethylenediaminetetraacetic acid (EDTA), 1% NP-40, 0.05% sodium deoxycholate, and 0.1% SDS) supplemented with protease inhibitors (10 μg/mL leupeptin, 20 μg/mL aprotinin, 1 mM phenylmethanesulfonyl fluoride, 1 mM NaVO_4_, and 100 mM NaF). Protein concentrations were determined using the Bradford protein assay (Bio-Rad Laboratories Srl., Segrate (MI), Italy). Cell extracts (50 μg) were resolved by SDS-PAGE (8%), transferred to polyvinylidene fluoride (PVDF) membranes, and probed with rabbit monoclonal anti-TRPM7 (1:1000, Abcam Ltd., Cambridge, UK), rabbit polyclonal anti-TRPM6 (1:500, Biorbyt Ltd., Cambridge, UK), rabbit polyclonal anti-tubulin or actin (1:1000, Sigma-Aldrich Srl., Milan, Italy) primary antibodies. Horseradish peroxidase-conjugated secondary antibodies (GE Healthcare Srl., Milan, Italy) were detected by use of the ECL Prime Western Blotting Detection Reagent (GE Healthcare Srl, Milan, Italy) and the ChemiDoc XRS system (Bio-Rad Laboratories Srl., Segrate (MI) Italy).Densitometric analysis was performed by using the National Institutes of Health (NIH) ImageJ software.

### 2.3. Mg^2+^ Influx Measurements

Subconfluent cells grown on 35-mm microscopy dishes (μ-dish, ibidi GmbH, Martinsried, Germany) were loaded with 3 μM Mag-Fluo-4-AM (Thermo Fisher Scientific, Monza (MI), Italy), and imaged in a Na^+^, Ca^2+^ and Mg^2+^-free buffer at a confocal laser scanning microscope, as previously described [19]. Cytosolic fluorescence signals were recorded as time series of 5 min at a sampling frequency of 30 frames/min. The baseline was monitored for 30 s, then MgSO_4_ was added drop wise to a final concentration of 20 mM. Changes in intracellular Mg levels at single cell level were estimated by the mean fluorescent increment ΔF/F [20]. Image analysis was performed by Leica Confocal Software on 10 representative cells in each microscopic field, and experiments were repeated independently at least three times.

### 2.4. Cell Cycle Analysis

Cells were fixed in 70% ethanol and stored at 4 °C until analysis. Prior to analysis, cell pellets were resuspended in 0.2 mg/mL of propidium iodide (PI) in Hank’s balanced salt solution containing 0.6% NP-40 and RNase (1 mg/mL). The cell suspension was then filtered and analyzed for DNA content on a Coulter EPICS 753 flow cytometer, as previously described [21]. The percentage of cells in different phases of the cell cycle was determined using ModFit analysis software (version 5.2, Verity Software House Inc., Topsham, ME, USA).

### 2.5. Scratch Assay

A scratch assay was performed as previously reported [18]. Cells were seeded in culture inserts (ibidi GmbH, 70,000 cells/well insert) and cultured for 24 h to allow attachment. Insert removal created a 500-μm-wide cell-free gap between two confluent cell monolayers. Wound closure was monitored with an Eclipse TE2000-S microscope (Nikon Instruments Spa, Campo Bisenzio (FI), Italy) for up to 48 h, and images were analyzed using the NIH ImageJ software. Results were expressed as the percentage of the initial gap area that was covered by cells after the indicated time.

### 2.6. Statistical Analysis

All experiments were repeated independently three times. Prism software (version 5.01, GraphPad Software Inc., La Jolla, CA, USA) was used for all statistical analyses. Statistical significance was evaluated using unpaired Student’s *t* test. Differences were considered statistically significant for a *p* value < 0.05, and significance levels were assigned as follows: * for *p* < 0.05, ** for *p* < 0.01.

## 3. Results

### 3.1. Cell Characterization

**Figure 1:**
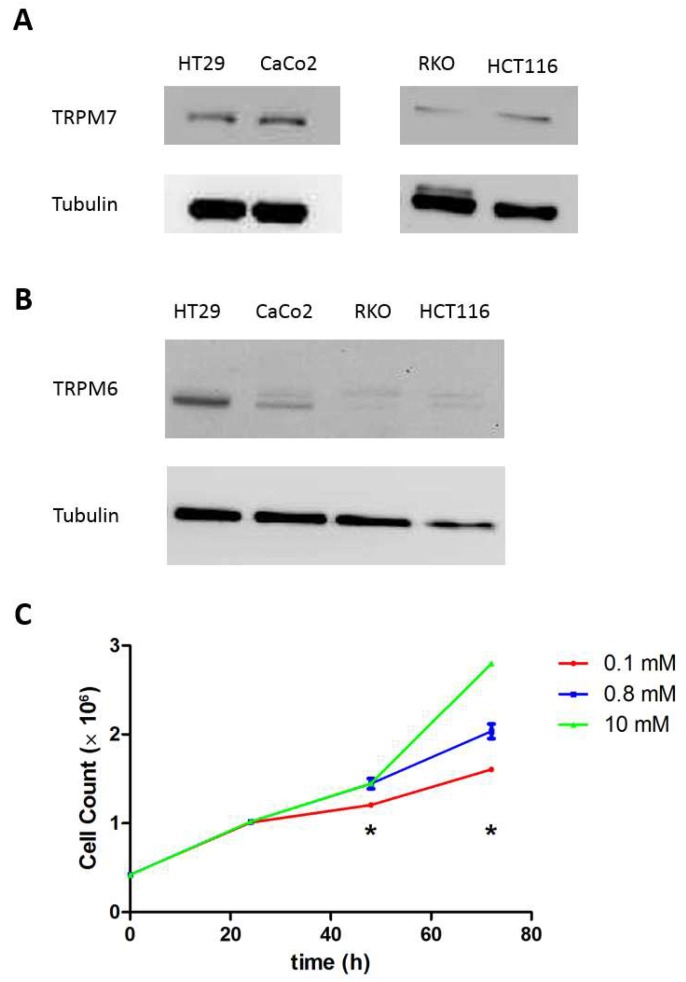
Human colon cells express both transient receptor potential melastatin type TRPM6 and TRPM7 and depend on extracellular Mg availability for growing. A representative Western blot (*n* = 3) is shown for (**A**) TRPM7 and (**B**) TRPM6 in a panel of human colon cell lines. Tubulin was used as a loading control. (**C**) HT29 cell proliferation in conditions of low (0.1 mM), normal (0.8 mM) and high (10 mM) Mg availability. Cells were grown in Mg-free Dulbecco’s modified Eagle’s medium (DMEM) supplemented as for routine culture plus the indicated amounts of MgSO_4_ and counted at 24, 48 and 72 h in duplicates (*n* = 3). * *p* < 0.05 by unpaired Student’s *t* test.

First, we assessed a panel of human colon cell lines (HT29, HCT116, RKO and Caco-2) for TRPM6 and TRPM7 protein expression using Western blot. As expected, all tested lines expressed both channels, though to various levels (Figure 1A,B). We focused on HT29 cells, which expressed the highest TRPM6 levels, but confirmed selected experiments in HCT116 cells. In HT29 cells, we also verified that proliferation was strictly dependent on extracellular Mg availability (Figure 1C).

**Figure 2:**
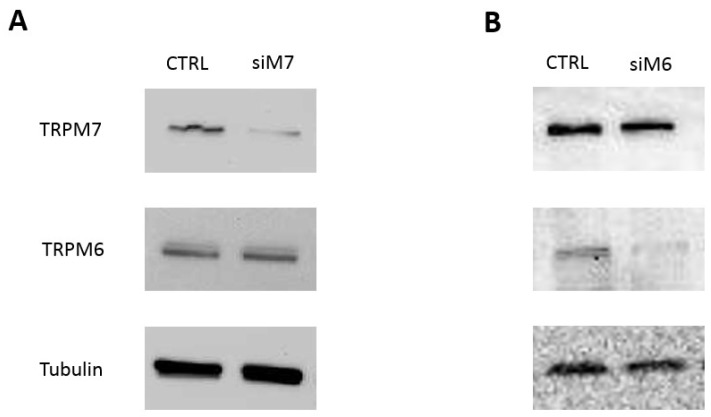
Specific short interfering RNA (siRNA) transfection efficiently downregulates TRPM7 and TRPM6 in human colon cells. HT29 cells were transfected with either (**A**) TRPM7 or (**B**) TRPM6-specific siRNA, and protein expression of both channels was evaluated by western blot 48 h after transfection. Non-silencing, scrambled sequences were used as controls (CTRL). Tubulin was used as a loading control. A representative blot is shown (*n* = 3). Note that TRPM7 silencing does not affect TRPM6 expression and vice versa. See Figure S1 in supplementary materials for complete blots, and Figure S2 in supplementary materials for mRNA expression by real time RT-PCR and additional data on HCT116 cells.

To downregulate specifically the expression of either channel, we used transient siRNA transfection and assessed mRNA and protein expression by real-time reverse transcriptase polymerase chain reaction (RT-PCR) and Western blot, respectively. Significant TRPM7 knock-down was achieved at the mRNA level and was confirmed at the protein level in both HT29 and HCT116 cells (Figure 2A, Figures S1 and S2). TRPM7 silencing did not affect TRPM6 expression (Figure 2A). Transfection of TRPM6-specific siRNA resulted in knock-down of TRPM6 expression with no effects on TRPM7 expression in HT29 cells (Figure 2B). Similar results were obtained in HCT116 cells (not shown).

These data prove that RNAi can achieve specific and significant downregulation of each channel and allow the distinguishing of the contribution of TRPM6 vs. TRPM7 to given cell functions.

### 3.2. Contribution of TRPM7 to Colon Cell Functions

**Figure 3:**
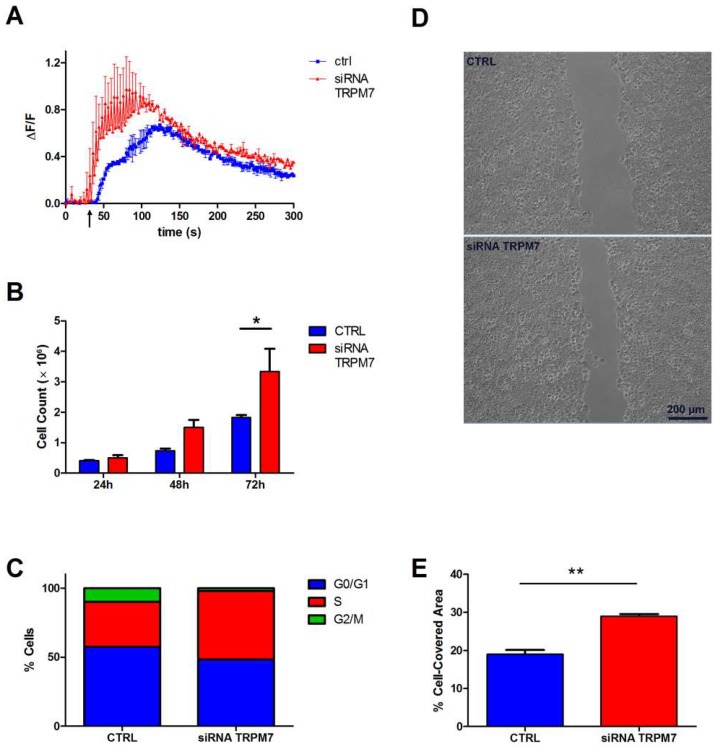
Contribution of TRPM7 to Mg^2+^ influx and Mg-dependent colon cell functions. HT29 cells were transiently silenced for TRPM7 as detailed in Materials and Methods. For HCT116 cells, see Figure S2. (**A**) Mg^2+^ influx capacity, as assessed 72 h after siRNA transfection. Mag-Fluo-4-loaded cells were challenged with 20 mM Mg (arrow) and time course of single-cell fluorescence was followed by live confocal imaging. The mean fluorescence (ΔF/F) of 10 cells ± standard error (SE) from a representative experiment is reported (*n* = 3). (**B**) Cell proliferation. Cells were counted at the indicated times after siRNA transfection; mean ± SE of three independent experiments is shown. (**C**) Cell cycle distribution, as assessed 72 h after siRNA transfection. The percentage of cells in each phase was evaluated by flow cytometry; results are from a representative experiment (*n* = 3). (**D**) Cell migration. Cells were silenced and grown to confluence in well inserts for 24 h. Insert removal created a 500-μm-wide cell-free gap, whose closure was monitored by microscopy. Representative images of the wound after 24 h in CTRL and siRNA TRPM7 cells are shown. (**E**) Cell migration. Quantification of the cell-covered area from three independent wound healing assays. Results are expressed as the percentage of the initial gap area that was covered by cells after 24 h. * *p* < 0.05, ** *p* < 0.01 by unpaired Student’s *t* test.

Next, we transfected cells with TRPM7-specific siRNA and evaluated the effects on cation influx and most closely related functions, such as proliferation and migration, in both HT29 and HCT116 cells. Results for HT29 cells are shown in Figure 3, while principal findings for HCT116 cells are reported in Figure S3. In preliminary experiments, TRPM7-silenced cells paradoxically exhibited a significant increase in constitutive divalent cation influx as assessed by the Mn^2+^ quenching technique (Figure S3A). Since the Mn^2+^ quenching assay does not discriminate the divalent species involved, to determine whether the transmembrane flux was due to an Mg^2+^ entry, we performed live imaging of cells loaded with the Mg-specific fluorescent probe Mag-Fluo-4. Fluorescence imaging confirmed that TRPM7-silenced cells had a higher Mg^2+^ uptake (Figure 3A). In addition, TRPM7-silenced cells showed a significantly faster proliferation rate, as assessed by cell counting (Figure 3B), and confirmed by a higher percentage of cells in the S phase of the cell cycle (Figure 3C). Finally, we performed a scratch assay to evaluate the effect of TRPM7 downregulation on wound healing capacity, which encompasses both proliferation and migration properties. We found that TRPM7-silenced cells closed the cell-free gap much more efficiently than control cells (Figure 3D,E).

Altogether, these results provide a consistent picture whereby downregulation of TRPM7 in TRPM6-expressing colon cells paradoxically results in strengthening of the characteristics usually associated to TRPM7 expression in other tissues, namely cation entry, proliferation and migration.

### 3.3. Contribution of TRPM6 to Colon Cell Functions

**Figure 4:**
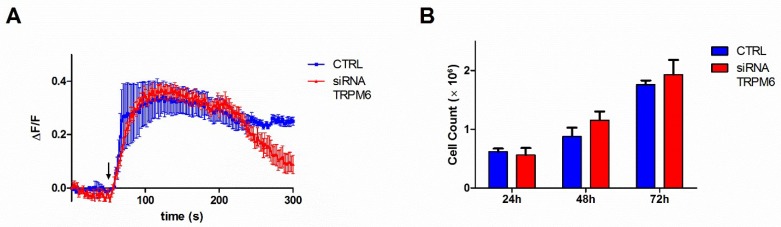
Contribution of TRPM6 to Mg^2+^ influx and cell proliferation in colon cells. HT29 cells were transiently silenced for TRPM6 and assessed at the indicate times after transfection. (**A**) Mg^2+^ influx capacity, as assessed 72 h after siRNA transfection. Mag-Fluo-4-loaded cells were challenged with 20 mM Mg (arrow) and time course of single-cell fluorescence was followed by live confocal imaging. The mean fluorescence (ΔF/F) of 10 cells ± SE from a representative experiment is reported (*n* = 3). (**B**) Cell proliferation. Cells were counted in duplicates; mean ± SE of three independent experiments is shown.

We moved on to examine TRPM6 contribution to the same cellular functions by transfecting a TRPM6-specific siRNA in HT29 cells. In marked contrast to TRPM7-silenced cells, downregulation of TRPM6 did not appear to affect significantly Mg^2+^ influx, as measured by Mag-Fluo-4 imaging (Figure 4A). Correspondingly, proliferation rate of TRPM6-silenced cells did not differ considerably from that of control cells (Figure 4B).

We conclude that partial downregulation of TRPM6 on a background of normal TRPM7 expression is not sufficient to alter significantly Mg^2+^ entry and cell proliferation.

### 3.4. Contribution of TRPM6/7 Channels

**Figure 5:**
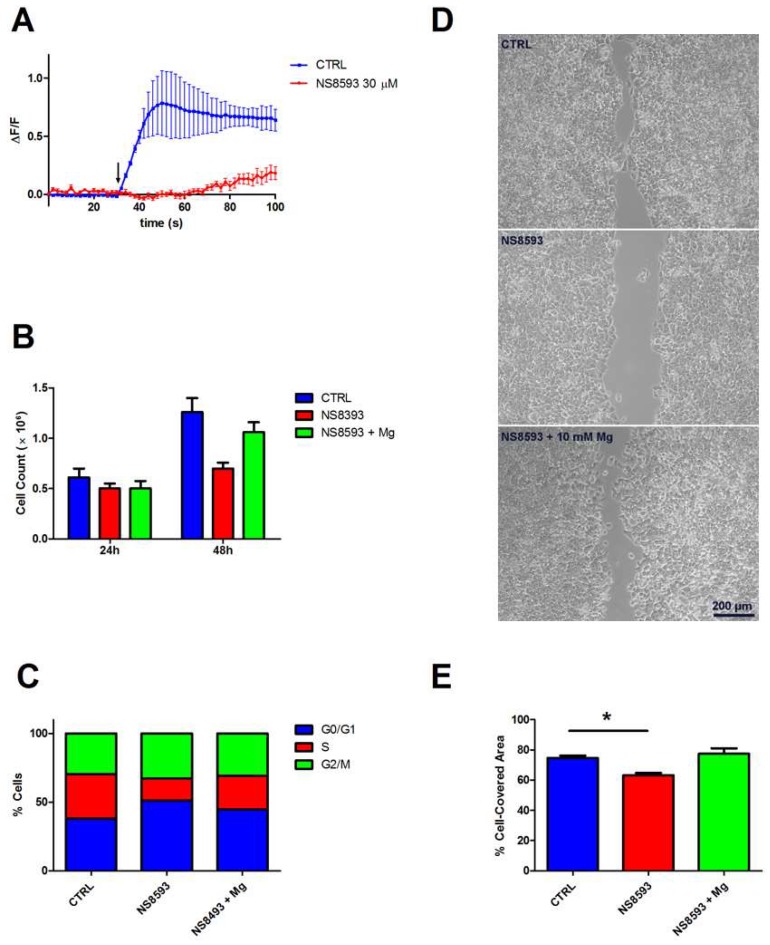
Contribution of TRPM6/7 channels to Mg^2+^ influx and cell proliferation. NS8593 was used at 30 μM. (**A**) Mg^2+^ influx capacity. Mag-Fluo-4-loaded cells were challenged with 20 mM Mg (arrow) in the presence of NS8593, and time course of single-cell fluorescence was followed by live confocal imaging. The mean fluorescence (ΔF/F) of 10 cells ± SE from a representative experiment is reported (*n* = 3). (**B**) Cell proliferation. Following NS8593 treatment, cells were counted at the indicated times; mean ± SE of three independent experiments is shown. (**C**) Cell cycle distribution. Percentage of cells in each phase was evaluated by flow cytometry 48 h after NS8593 treatment. Results are from a representative experiment (*n* = 3). (**D**) Cell migration. Cells were grown to confluence in inserts that were subsequently removed leaving a 500-μm-wide cell-free gap; NS8593 was added 4 h before insert removal. Wound closure was monitored by microscopy; representative images of the wound after 48 h in CTRL and NS8593-treated cells are shown. (**E**) Quantification of the cell-covered area (*n* = 3); results are expressed as the percentage of the initial gap area that was covered by cells after 48 h. * *p* < 0.05 by unpaired Student’s *t* test.

In the light of the results reported in the previous sections, we hypothesized that the non-redundant role of TRPM6 and TRPM7 in colon cells might be due to the formation of heteromeric channels. It is known that, in cells expressing both TRPM6 and TRPM7, the functional ion channel at the plasma membrane may consist of both TRPM7 homomers and TRPM6/7 heteromers [5]. To support the idea that Mg^2+^ influx through TRPM6/7 channels is critical for colon cell function, we used pharmacological inhibition by NS8593, a specific TRPM6/7 inhibitor [22,23]. In the presence of 30 μM NS8593, colon cells exhibited a reduced Mg^2+^ influx capacity, as evidenced by a markedly delayed and slower uptake kinetics (Figure 5A). Correspondingly, NS8593-treated cells had a decreased proliferation rate (Figure 5B), and a lower percentage of cells in the S phase of the cell cycle (Figure 5C). Furthermore, NS8593 significantly inhibited wound healing capacity (Figure 5D,E). Importantly, inhibition by NS8593 on cell proliferation and migration was successfully rescued by Mg^2+^ supplementation (10 mM).

Therefore, we conclude that in colon cells Mg^2+^ uptake and related cellular functions strictly depend on formation of TRPM6/7 heteromers, which are the physiological active form of the channel.

## 4. Discussion

In the present paper, we report the molecular characterization of Mg^2+^ uptake in colon cell lines and demonstrate that the presence of the TRPM6 channel is essential to guarantee intestinal Mg absorption and Mg-dependent epithelial functions. Surprisingly, we show that TRPM7 downregulation resulted in increased Mg^2+^ influx, and consequently faster cell proliferation and migration of colon cells (Figure 3). This is opposed to a vast body of literature indicating that in several other cell types TRPM7 expression is positively associated with proliferation and migration, in particular in tumor cells [24,25]. The most straightforward explanation for an increased Mg^2+^ influx in the face of a decreased TRPM7 expression would be that TRPM7 downregulation triggered an up-regulation in other Mg-transporting proteins, as previously demonstrated in different models [26,27]. Although we cannot rule out the involvement of still undisclosed players, we have convincing evidence that TRPM7-silenced cells displayed unchanged expression of the two major candidates, i.e., TRPM6 (Figure 2A) or MagT1 (Figure S1B). In contrast to TRPM7 knocking-down, TRPM6 silencing did not appear to affect significantly Mg^2+^ uptake and proliferation in colon cells (Figure 4).

To explain the apparent paradox in our results, we must take into account the coexistence of significant levels of both TRPM6 and TRPM7 in all tested cells. This implies that the functional ion channel at the plasma membrane may consist of both TRPM7 homomers and TRPM6/7 heteromers. The prevalence of either form may depend on the expression ratio between the two proteins, and may result in different functional properties.

It was originally proposed that native TRPM6 functions primarily as a subunit of heteromeric TRPM6/7 complexes [15], and this model has been corroborated by recent data [14,23,28]. The emerging picture is that TRPM6 and TRPM7 differentially contribute to regulatory characteristics of the heteromeric TRPM6/7 channel, so that the activity of the complex will hardly be affected by physiological intracellular concentrations of Mg^2+^ and Mg-ATP [23] or by osmotic changes [28]. This mechanism appears to be an indispensable prerequisite for efficient transcellular Mg^2+^ transport in intestinal cells, where a high and constant Mg^2+^ uptake should be uncoupled from cellular metabolism of Mg^2+^ and Mg-ATP [23], and should remain unaffected by frequent osmotic changes [28]. Such a functional fingerprint is probably not required in other cell types, which indeed only express TRPM7.

**Figure 6:**
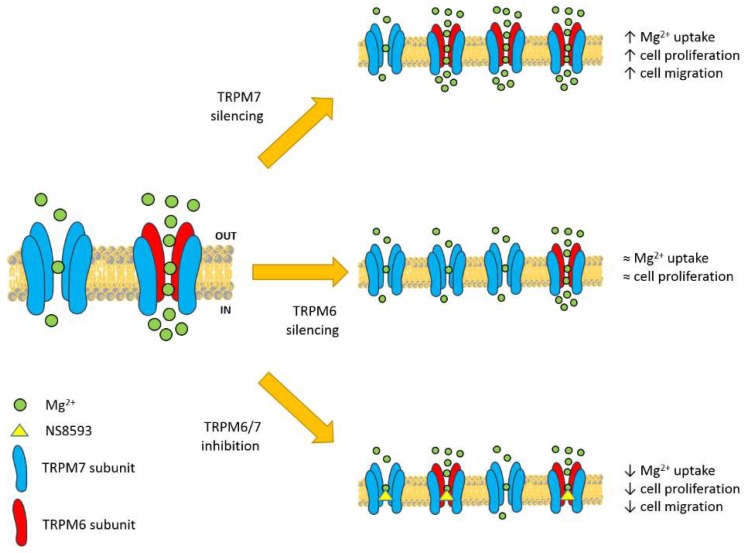
Suggested model for the role of TRPM6 in intestinal Mg absorption. Colon cancer cells express both TRPM7 and TRPM6, which can assemble into functional homomers or heteromers at the plasma membrane (left). The different regulation of TRPM7 and TRPM6/7 channels in physiological conditions determines higher Mg^2+^ fluxes through TRPM6/7 heteromers. Thus, the ratio between differently assorted tetramers affects cation influx and related signaling pathways (right panels). Partial TRPM7 silencing favors formation of TRPM6/7 channels, thereby increasing Mg^2+^ influx, and consequently cell proliferation and migration (upper right panel). In turn, partial TRPM6 knocking-down promotes assembly of TRPM7 homomers, which sustain basal Mg^2+^ influx and proliferation (middle right panel). Pharmacological inhibition of assembled channels decreases overall Mg^2+^ influx, and downstream functions (lower middle panel).

Our data fit perfectly in this model: Silencing either TRPM7 or TRPM6 is in fact a way to alter the relative abundance of the two proteins, and consequently will affect the ratio between differently assorted tetramers in favor of TRPM6/7 heteromers or TRPM7 homomers, respectively (Figure 6). We propose that TRPM7 downregulation tips the balance towards increased relative abundance of TRPM6/7 complexes, which are responsible for the observed increase in Mg^2+^ influx and Mg-dependent cell functions. Accordingly, in electrophysiological measurements current amplitudes of TRPM6/7 complexes were found to be higher than those of TRPM7 homomers [15,23,29]. In contrast, TRPM6 downregulation should favor TRPM7 homomerization and result in overall reduced Mg^2+^ influx and cell proliferation. Although we did not find a remarkable effect of TRPM6 downregulation (Figure 4), it must be noted that we only achieved a partial TRPM6 downregulation, which may be compatible with retaining sufficient levels of TRPM6/7 channels to foster Mg^2+^ entry and cell proliferation. However, when we used NS8593 as a potent and specific way to block channel activity regardless of its composition, we did obtain the expected reduction in Mg^2+^ uptake and cell growth (Figure 5). Furthermore, the observed inhibition was rescued by Mg supplementation, which proves that as long as sufficient Mg^2+^ entry occurs, cellular Mg homeostatic mechanisms are able to sustain cell proliferation and migration even with reduced TRPM6/7 function(s). Altogether, our findings confirm that TRPM6/7 complexes and TRPM6/7-mediated Mg^2+^ influx are absolutely necessary in human epithelial colon cells. More specifically, TRPM6 has an indispensable role in controlling Mg^2+^ entry and cell proliferation, and other ion channels, including the highly homologous TRPM7 channel, cannot replace its function.

The main strength of our work is that we carried out our functional characterization in a completely naïve cell model, without resorting to heterologous expression systems. In the past, overexpression of recombinant proteins has greatly contributed to investigating TRPM6 and TRPM7 currents and regulation, but also generated conflicting and still unexplained results regarding TRPM6 [5]. Our results are limited to few prototypal human colon cell lines, and need further investigation before they can be generalized. In particular, the proof of concept of our hypothesis would require determination of the absolute expression levels of TRPM7 and TRPM6 as well as of the exact stoichiometric architecture of TRPM6/7 heteromers, which is technically very challenging.

Despite its limitations, our interpretation is completely in line with wider and more sophisticated studies demonstrating that TRPM6 is essential for intestinal magnesium absorption and systemic Mg balance. [14]. As for TRPM7, to the best of our knowledge, no studies investigated either mineral homeostasis in TRPM7-deficient adult mice or specific ablation of TRPM7 in the intestine. However, we would not expect to reproduce our results in a TRPM7 KO system, because the complete absence of TRPM7 would also impair TRPM6 proper localization and function [14,15]. Interestingly, heterozygous TRPM7 knock-in mice devoid of the kinase domain or activity display an altered systemic Mg homeostasis [7,30]. Although relative levels of TRPM7 homomers vs. TRPM6/7 heteromers might change in heterozygous mice, this situation cannot be exactly matched with our working model, since the current thinking is that the TRPM7 kinase moiety may function as a sensor of the organismal Mg status [30]. Overall, we cannot compare our in vitro data with any existing model. Nevertheless, the peculiarity, and paradoxically, the strength, of our model is that we only partially knock-down expression of either channel, thereby modulating the assortment of the functional complexes on a background of concurrent TRPM6 and TRPM7 expression.

## 5. Conclusions

In conclusion, our data confirm the existing view that maintenance of systemic Mg homeostasis requires a constant Mg supply that can be warranted only by TRPM6/7 heteromers in the intestine, and provide a simple and effective model to investigate the functional relationship between TRPM6 and TRPM7. 

## Supplementary Materials

The following are available online at http://www.mdpi.com/2072-6643/10/6/784/s1. Figure S1: Complete blots corresponding to the images shown in Figure 2A. Figure S2: Transient siRNA transfection efficiently downregulates *TRPM7* mRNA in human HT29 and HCT116 colon cells (**A**), and TRPM7 protein in HCT116 cells (**B**). Figure S3: Contribution of TRPM7 to Mg^2+^ influx and Mg-dependent cell functions in HCT116 cells. (**A**) Mn^2+^ quenching; (**B**) Mg^2+^ influx capacity; (**C**) Cell Proliferation; (**D**) Cell cycle distribution.
