# Supplementary material for: TRPM6 is Essential for Magnesium Uptake and Epithelial Cell Function in the Colon
Source: Nutrients. 2018 Jun 18;10(6):784. doi: 10.3390/nu10060784 (PMC6024373; doi:10.3390/nu10060784)
Supplement: Supplementary file 1 [file nutrients-10-00784-s001.pdf]

Supplementary Information

1.Supplementary Methods

1.1 Mn^{2+} Quenching Assay

Cells were grown on glass cover slips in 35-mm dishes at a density of 8×10^4 cells. After 48h, cells were loaded in cell growth medium at 37°C for 1h with 3 µM of Fura 2-AM (Invitrogen), rinsed three times and transferred to a perfusion chamber on a Zeiss microscope equipped for fluorescence. Cells were perfused for 1 min with extracellular solution containing (in mM) 145 NaCl, 5 KCl, 2 $CaCl_2$, 0.4 $MgCl_2$, 10 HEPES and 5 glucose (pH adjusted to 7.4 with NaOH). Subsequently, Ca^{2+} was replaced by Mn^{2+} (2 mM). Fura-2 fluorescence was excited at 360 nm with a monochromator (TILL® Photonics, Munich, Germany), and emission was monitored at 510 nm by a CCD camera coupled to a Zeiss inverted microscope (Carl Zeiss MicroImaging, LLC, Oberkochen, Germany). After Mn^{2+} perfusion, Fura-2 fluorescence described a linear decay, whose slope is correlated with the rate of Mn^{2+} influx. The slope was calculated by subtracting the slope of Fura-2 fluorescence obtained in basal conditions (culture conditions) and after Mn^{2+} application.

1.2 Real Time RT-PCR

RNA extraction was performed using the standard Trizol-phenol-chloroform protocol. Total RNA (1 µg) was reverse-transcribed into cDNA with random hexamers and MultiScribe Reverse Transcriptase (Applied Biosystems). Real time RT-PCR was performed on a LightCycler system (Roche) using a mix containing SYBR green (Applied Biosystem). TRPM7-specific primers were: forward 5′-GTCACTTGGAAACTGGAACC-3′ and reverse 5′-CGGTAGATGGCCTTCTACTG-3′. β-actin-specific primers were: forward 5′-CAGAGCAAGAGAGGCATCCT-3′ and reverse 5′-ACGTACATGGCTGGGGTG-3′. TRPM7 mRNA quantities were normalized to β-actin as a housekeeping gene.

2. Supplementary Figures

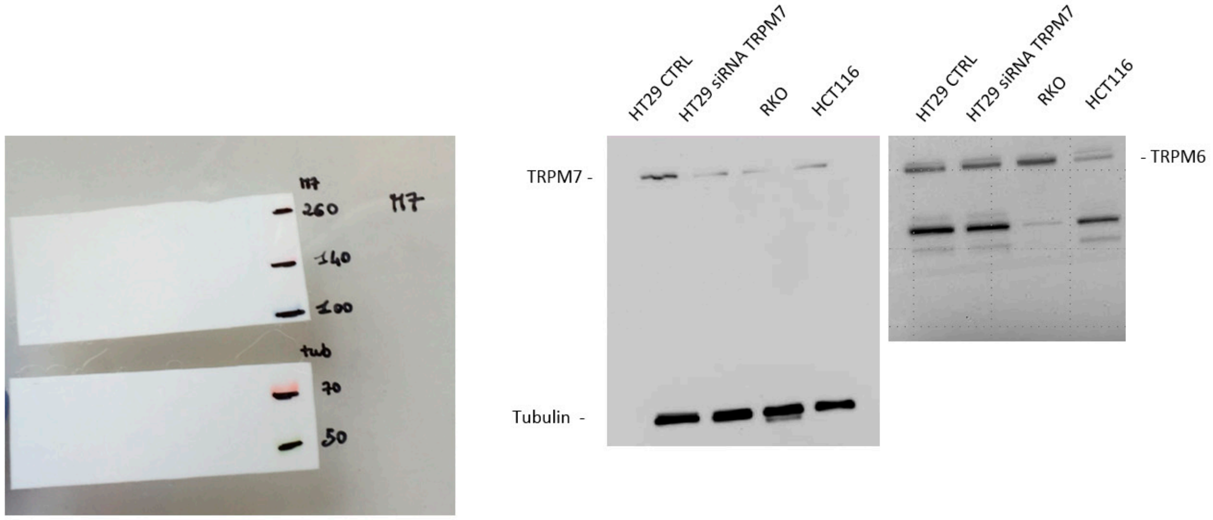

Figure S1. Complete blots corresponding to the images shown in Figure 2A

A

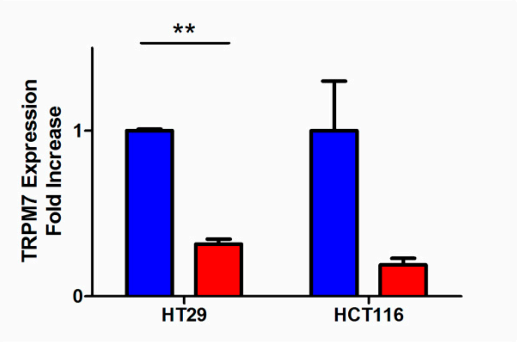

B

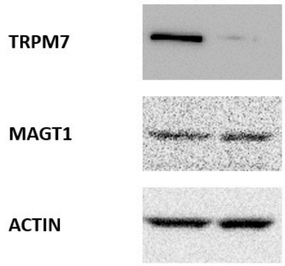

HCT116

Figure S2. Transient siRNA transfection efficiently downregulates A) *TRPM7* mRNA in human HT29 and HCT116 colon cells, and B) TRPM7 protein in HCT116 cells. Note that TRPM7 silencing does not affect MAGT1 expression.

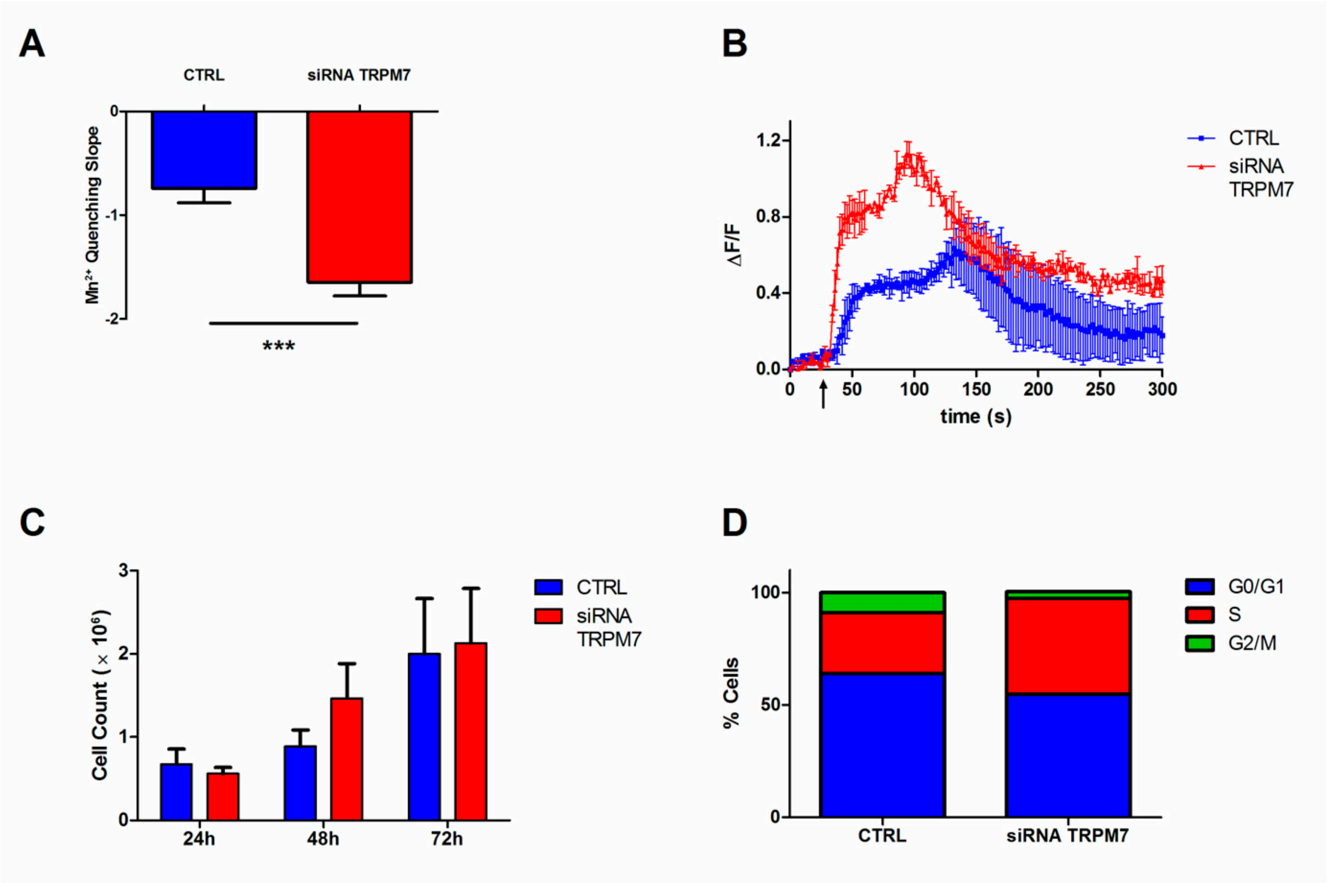

Figure S3. Contribution of TRPM7 to Mg^{2+} influx and Mg-dependent cell functions in HCT116 cells. A) Mn^{2+} quenching. B) Mg^{2+} influx capacity. C) Cell Proliferation. D) Cell cycle distribution.
